# Exploring Naturalistic Diffusion of an Evidence-Based Mental Health Intervention across Peer Networks of Youth in Sierra Leone

**DOI:** 10.3390/ijerph20054059

**Published:** 2023-02-24

**Authors:** Alethea Desrosiers, Laura Bond, Morgan Hoffman, Praveen Kumar, Carolyn Schafer, Isha W. Metzger, Alpha Vandi, Miriam Hinton, Theresa S. Betancourt

**Affiliations:** 1 Department of Psychiatry and Human Behavior, Brown University, Providence, RI 02906, USA; 2 School of Social Work, Boston College, Chestnut Hill, MA 02496, USA; 3 Institute for Public Health and Medicine, Northwestern University, Evanston, IL 60208, USA; 4 College of Arts and Sciences, Georgia State University, Atlanta, GA 30302, USA; 5 Caritas-Freetown, Freetown, Sierra Leone

**Keywords:** diffusion, youth, mental health, behavioral interventions, adjustment, resilience

## Abstract

Background: Understanding the mechanisms by which evidence-based interventions (EBIs) for mental health are naturally diffused among youth in low-and middle-income countries—particularly those with histories of violence and civil unrest—can illuminate which intervention elements are most transferrable and inform scale-up decisions that support youth adjustment. This study explored the diffusion of an evidence-based mental health intervention—the Youth Readiness Intervention (YRI)—among peer networks of Sierra Leonean youth (aged 18–30) who participated in a trial of the intervention as integrated into youth entrepreneurship programs. Methods: Trained research assistants recruited index participants who had completed the YRI integrated within entrepreneurship training (N = 165) and control index participants (N = 165). Index participants nominated three of their closest peers. Nominated peers were recruited and enrolled in the current study (N = 289). A sub-sample of index participants and peers participated in dyadic interviews (N = 11) and focus group discussions (N = 16). Multivariate regression analysis compared YRI knowledge levels among YRI participants’ peers relative to control participants’ peers. Results: Qualitative findings supported the diffusion of several YRI skills and components across peer networks (i.e., progressive muscle relaxation and diaphragmatic breathing). Quantitative findings indicated that YRI knowledge was significantly higher for YRI participants’ peers (β = 0.02, *p* < 0.00) compared to control participants’ peers. Conclusion: Findings suggest that diffusion of evidence-based intervention components can occur naturally among peers in post-conflict LMIC settings. Developing tools to promote the diffusion of the most transferrable EBI components across peer networks could help maximize the benefits of mental health interventions for youth adjustment and resilience in post-conflict settings.

## 1. Introduction

More than one billion children and youth live in countries affected by war and communal violence [1]. The consequences of exposure to violence, poverty, and personal loss include high rates of depression, anxiety, and post-traumatic stress symptoms among youth who reside in post-conflict settings throughout the world, including in Sub-Saharan Africa [2,3,4]. Exposure to compounded adversity and poor mental health can also increase the odds of suicidal behaviors and negatively impact positive developmental outcomes, thus limiting the ability of youth to engage successfully in education, employment, and other life opportunities [5,6,7]. Although there are many evidence-based mental health interventions with demonstrated effectiveness, few are delivered with sufficient breadth to benefit large populations and large geographic areas, and even fewer are disseminated in LMICs [8,9]. There are many barriers to scaling up and sustaining evidence-based mental health interventions, particularly in LMICs with histories of violence and loss; thus, access to evidence-based interventions for vulnerable youth remains limited, and the mental health treatment gap remains high [10,11]. Innovations are needed to improve access to and utilization of evidence-based mental health services that support positive youth adjustment in Sierra Leone and other LMICs with histories of violence and loss.

In Sierra Leone, a nation in West Africa with a long history of compounded adversity (e.g., civil war, Ebola epidemic, recurrent mudslides), the mental health treatment gap has been estimated at 98% in a 2009 survey [12]. However, this estimate was obtained from a survey in 2009, and there have been no subsequent studies on the prevalence of mental health disorders in Sierra Leone. Until 2012, the only treatment facility for mental health disorders was the Sierra Leone Psychiatric Hospital. In 2012, 21 nurses were trained in the mental health gap action plan (mhGAP), and in 2015, 15 nurse-led mental health units were established across 13 districts. Additional services for mental health problems have not been available [13]. Within this context, it is especially critical to consider innovative strategies for increasing the provision and reach of mental health services in Sierra Leone.

Understanding the naturalistic diffusion of skills learned during participation in evidence-based mental health interventions, including why and how core components of interventions are diffused across peer networks, could be a critical innovation to address barriers to the implementation and sustainment of evidence-based interventions among youth in Sierra Leone and other LMICs. *Diffusion* is the process of untargeted and unplanned spread of new practices over time among network members of a social system, including peer networks [14]. Unpacking the mechanisms through which evidence-based interventions are naturally diffused across peer networks, including whether specific characteristics of personal networks facilitate diffusion, could not only illuminate which intervention elements are most transferable but also help inform delivery and scale-up decisions. Furthermore, pinpointing which components are more likely to be diffused across social networks may guide targeted adaptations for implementation in other LMICs and post-conflict settings to better support youth adjustment and resilience at minimal costs.

Prior research on the diffusion of evidence-based interventions across social networks in the United States suggests that several social network characteristics are associated with the diffusion of mental health intervention effects among youth over time [15]. These characteristics include *representativeness*, or the degree to which an intervention participant is similar to the broader population with respect to important demographic or behavioral characteristics, and *social integration*, or the extent to which networks are tightly interconnected. However, the diffusion of mental health interventions has not yet been studied in Sub-Saharan Africa and/or other post-conflict settings. A small number of studies focused on other health topics in Sub-Saharan Africa support the feasibility of collecting data on personal networks [16,17,18]. Personal networks focus on the structure and composition of connections from the perspective of a single individual, whereas social networks focus on the interconnections of all individuals in a social group [19]. Given that informal peer support has been positively related to mental health and well-being [20,21], harnessing personal network characteristics that facilitate the peer-to-peer transfer of adaptive behaviors could further promote mental health across a wider segment of the population. Further research is needed to understand whether personal network characteristics might influence the diffusion of mental health interventions among youth in Sub-Saharan Africa and other post-conflict settings and how these network characteristics can be capitalized on to actively spread mental health interventions that promote resilience in these contexts.

The current study builds upon a hybrid implementation effectiveness trial of an evidence-based mental health intervention, the Youth Readiness Intervention (YRI), integrated within entrepreneurship training for vulnerable youth in Sierra Leone. The YRI was developed using community-based participatory research methods to address the unmet mental health needs of war-affected Sierra Leonean youth [22]. The YRI is a group-based intervention that integrates core components of cognitive behavioral and interpersonal therapy and can be feasibly delivered by a wide range of lay workers (see Supplementary Table S1). Intervention development was informed by extensive mixed methods research, including a prior longitudinal study with youth affected by conflict, qualitative interviews with community stakeholders, and engagement with a community advisory board [22]. All YRI components were culturally and contextually adapted and reviewed by the community advisory board. The YRI has demonstrated effectiveness in improving mental health, psychological adjustment, and daily functioning among Sierra Leonean youth facing adversity, and youth have reported high levels of satisfaction with the intervention [23,24]. In prior research, YRI participants also reported sharing YRI skills and components (i.e., deep belly breathing) with their friends, suggesting that naturalistic diffusion of YRI techniques has the potential to occur across communities and social networks [25].

The current study explored the process of naturalistic diffusion of YRI components between youth who have participated in the intervention and their peers. Rogers’ *Diffusion of Innovations Theory* provides a useful framework for exploring the adoption of evidence-based interventions such as the YRI. According to this theory, diffusion is the process of communicating an innovation through specific channels over time among network members of a social system and investigating the channels and patterns of communication within peer social networks [14,26]. Innovation is defined as an idea or project that is considered new by an individual [14]. This study illuminates how youth receiving the YRI share certain YRI components with their peers and determines which components are most transferrable. For example, youth may share YRI components through knowledge transfer (e.g., demonstration) or through persuasion (e.g., sharing personal testimonies). Additionally, drawing from the *Dynamic Sustainability Framework*, which conceptualizes sustainability as an “adaptation phase” that integrates and institutionalizes interventions within local organizational cultural contexts [27], understanding the diffusion of the core YRI components (psychoeducation, cognitive restructuring, emotion regulation, behavioral activation, problem-solving, interpersonal skills) can inform an adaptation phase in which the most salient and transferrable components can be repackaged and integrated into new settings and platforms.

More specifically, this study investigated mechanisms of diffusion of an evidence-based mental health intervention—the Youth Readiness Intervention (YRI)—among YRI participants’ peer networks of Sierra Leonean youth (aged 18–30) who participated in the intervention. We examined (a) which components are diffused, (b) how they are diffused (i.e., demonstration, modeling, persuasion), and (c) with whom they are most likely to be shared with. We also explored (d) whether YRI participants’ peers demonstrate greater knowledge of YRI skills and components than control participants’ peers and (e) whether personal network characteristics are associated with the uptake of YRI skills and techniques. We hypothesized that the YRI components perceived as simplest to use would be most commonly shared among peer networks and that the more complicated techniques would be less shared. We also hypothesized that youth would be more likely to share techniques with same-gender peers and to share these techniques through conversations and demonstrations of basic skills. Finally, we hypothesized that YRI participants’ peers would report greater knowledge of YRI components than control participants’ peers.

## 2. Methods

### 2.1. Participants

We recruited index participants who had enrolled in a hybrid-implementation effectiveness trial of the YRI implemented with entrepreneurship training (Youth FORWARD/U19 MH109909) for youth aged 18–30. We obtained informed consent from index participants who had completed the YRI delivered within entrepreneurship training (N = 165) and control index participants (N = 165; N = 330 total) and enrolled them in the current study. We applied an egocentric fixed choice design [28], which samples a population of interest and collects information on each respondent and their contacts, resulting in a collection of egocentric networks.

Following this design, index participants who enrolled in the current study completed a standard ego network survey in which they nominated three of their closest peers. Nominated peers of both YRI and control index participants were then recruited and enrolled in the current study (N = 879). *Inclusion criteria* were (a) being a part of a YRI participant’s social network and (b) being male or female aged 18 or over. *Exclusion criteria* were (a) being a current YRI participant or (b) exhibiting severe, active suicidality or psychosis as assessed via the MINI-SCID diagnostic assessment administered by a study social worker. All peer participants provided informed consent to participate in the study.

Participants were located across 3 rural districts (Kono, Koinadugu, and Kailahun) and 16 chiefdoms in the Eastern province of Sierra Leone. Kailahun is the largest district of the three, with a population size of 465,048 and a district-level poverty rate of 60.9%. Kono has a population of 242,722, and Koinadugu’s population size is 335,471, with 61.5% and 54% district-level poverty rates, respectively [29]. In Sierra Leone, the designation of “rural” is generally related to a lower population density than in urban areas, and agricultural and informal work is the primary means of income generation. The geographical proximity of households may be more spread out in rural regions, but there can also be an increased sense of social connectedness in the smaller villages.

### 2.2. Procedures

#### 2.2.1. Quantitative Data Collection Methods

Baseline quantitative data on peers were collected from September 2019 through December 2019. Follow-up data were collected between November 2020 and February 2021. Attrition was 5.33%, with 45 (5.11%) peers being lost to follow-up. The main causes for attrition were difficulties in locating participants. If nominated peer participants were listed by more than one Youth FORWARD index participant, these peers were not treated as duplicated records if they actually pertained to two different ego networks.

#### 2.2.2. Quantitative Measure of YRI Skills and Knowledge

YRI Skills and knowledge levels were assessed with a 24-item multiple-choice scale developed specifically to evaluate knowledge of YRI concepts, skills, and techniques (i.e., diaphragmatic breathing, progressive muscle relaxation problem-solving skills, communication skills, goal setting). Peers completed this measure at follow-up data collection. An example item is the following: “the steps of sequential problem solving include: (a) identify the problem; (b) brainstorm solutions; (c) identify consequences; (d) all of the above.” Items are scored as correct (1) or incorrect (0), and an average is calculated to reflect the YRI skills and knowledge score.

#### 2.2.3. Qualitative Data Collection Methods

A subset of YRI index participants and their peers were selected based on a multivariate sampling matrix to participate in qualitative interviews to explore the diffusion of YRI skills and components among peers. Table 1 displays demographic information for qualitative interview participants. A total of 22 semi-structured key informant interviews were conducted with dyads of YRI participants and their selected peers at two time points, first in December 2019 and later in December 2020. Eight focus group discussions were also conducted at two time points, first in September 2019 and later in January 2021. Dyadic interviews and the focus group discussions explored how, if at all, YRI participants shared specific YRI skills and components with their peers; what YRI participants shared, if anything, and what was easier or harder to share; whom YRI participants shared skills and components with, if anyone; and finally, how peers of YRI participants perceived the sharing of skills and components (i.e., whether they were able to comprehend and utilize these skills in their own lives). Both key informant interviews and focus group discussions used a semi-structured interview guide with questions, and potential follow-up prompts. At the second time point, additional questions were added regarding any changes in sharing and/or use of YRI skills and components.

All interviews were audio recorded by trained research assistants who were fluent in both English and Krio. After data collection, research assistants transcribed each interview and translated them into English.

#### 2.2.4. Ethical Considerations

Trained research assistants read consent forms aloud, explained the risks of involvement to participants, and asked if participants had any follow-up questions. For focus group participants, research assistants explained that all study participants would be asked to keep everything shared in the discussion confidential; however, this could not be guaranteed by the research team. If participants agreed to take part in the study, informed consent was obtained both verbally and in writing via a signature or fingerprint. The study was approved by the Boston College Institutional Review Board and the Sierra Leone Ethics and Scientific Review Committee.

### 2.3. Data Analysis

#### 2.3.1. Quantitative Data Analysis

Models involving network measures and respondent characteristics are more holistic and can enhance our understanding of the social context [30,31]. In personal network analyses, the nature and pattern of the immediate personal relationships of the respondents (YRI participants or control participants) and their network members (called peers) are investigated. The key components of personal networks include the following: (1) a focal node (respondent), (2) peers reported by the respondents, and (3) the ties between the respondent and their peers [3]. In this study, we deployed a trained enumerator who led the personal network data collection in the field. We adapted and abridged PERSNET, a standard personal network survey instrument routinely used to collect personal network data [30,31,32,33,34,35]. The PERSNET was administered to the YRI and control index participants who had consented and enrolled in the study.

We adjusted for four compositional measures of personal network characteristics routinely used along with demographic characteristics to explain the diffusion of innovation in numerous studies [30,31,32,33,34,35,36]. We also adjusted for the following demographic variables: education (i.e., never attended, primary, junior secondary, and senior secondary), gender, and relationship status (i.e., partner, no partner). The compositional measures are described below:

1. Proportion homophily of gender: This is defined as the proportion of peers’ who are of the same gender as that of the corresponding YRI or control participants. In terms of gender, this measure interprets the proportion of peers of the same gender as that of the respondent. This measure explores the extent of similarity or parity between the gender of the respondents and their nominated peers. For instance, if a female respondent reports three peers with genders: female, male, and male, then the proportion homophily of gender is 1/3 or 0.33.
2. Gender diversity in peers: This measure evaluates the heterogeneity of gender in the peers for each of the respondents. Based on the recommendations of our cultural and field experts, we solicited two options for the gender (female/male) of the peers from our respondents. We calculated the gender diversity in peers using Blau’s index formula: $1-\sum_{k}{\left(p_{k}\right)}^{2}$ [32,36]. In this formula, *p* is the proportion of respondents’ peers in category *k.* For instance, assume a respondent reports three peers with genders: females, males, and males; then, the gender diversity in peers is 0.44. Gender diversity is a peer-only measure. It does not account for the gender of the respondent.
3. Relationship diversity: This measures the heterogeneity in the nature of relationships between the respondent and the corresponding peers. In this study, we prompted the respondents to elicit their relationships with the reported peers from the following five options: community leader, coworker, friend, neighbor, and other. We then calculated the relationship diversity using Blau’s index formula (discussed above) [32,36].
4. Diversity in residential distance: This measures the variation in the residential distance of the peers from the respondents’ residences. In this study, we prompted the respondents to elicit the residential distance of the peers from the following three options: (1) same house, (2) 0–5 miles, (3) more than 5 miles. We calculated diversity in the residential distance accordingly using Blau’s index formula (discussed above) [32,36].

We conducted a multivariate regression analysis to compare the difference in the YRI skills and knowledge among the peers of YRI participants relative to the peers of control participants at the follow-up time point. Models included personal network characteristics of the YRI and control participants, the gender of reported peers, and the demographic measures of the YRI and control participants. Multiple peers were clustered in index participants (YRI or control). We clustered the model by index participants. The models used a 95% confidence interval and a *p*-value of 0.05 for significance. We used Stata version 17 for regression analyses.

#### 2.3.2. Qualitative Thematic Analysis

Two authors used an inductive, grounded theory approach to data analysis guided by the research questions and the Boyatzis approach to codebook development [37]. The first step was “open coding” to read through each transcript in-depth, while taking notes and writing memos on emerging themes. Then, authors began developing a codebook based on notes and memos taken. Codebook development was an iterative process in which the codebook was tested on a subset of transcripts and then improved as necessary. In line with the Boyatzis approach, codebooks contained three levels of codes, code definitions, and inclusion and exclusion criteria for each code. After the codebook was finalized, authors established inter-rater reliability between each other and divided the transcripts for coding (79% agreement with minimum of 50% overlapping). Throughout the process of data analysis, authors met weekly to discuss the themes emerging in the data, refine the codebook, and decide together how to code any challenging sections of the transcripts. All qualitative analysis utilized MAXQDA software [38].

## 3. Results

### 3.1. Quantitative Analysis

The average age of index participants was 25.04 (sd: 3.52), and 46.36% were female (53.64% male). Regarding educational status, 22.77% never attended school, 21.85% completed primary education, 31.08% completed junior secondary, and 24.31% completed senior secondary education. For peers, the average age of nominated peers was 27.77 (sd: 6.00), and 44.8% were females (55.16% males). Most index participants described the nature of the relationship with peers as “friendship” (85.3%).

The results of multivariate regression analyses are presented in Table 2. Both the unadjusted and adjusted models indicated that the YRI skills and knowledge were significantly higher for peers of YRI participants (β = 0.02, *p* < 0.05) compared to peers of control participants.

**Figure 1:**
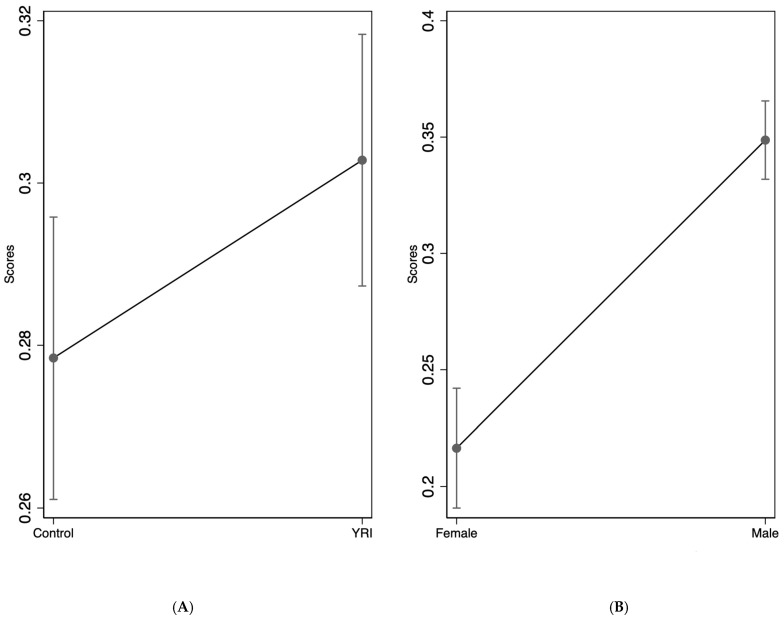
YRI skills and knowledge scores by YRI participation and gender of peers. (**A**) study arm, (**B**) Gender of the Peers.

The results also demonstrated that the gender of peers does not moderate the association between the index participants’ study arm (e.g., YRI or control) and the YRI skills and knowledge scores of peers. No personal network characteristics were significantly associated with YRI skills and knowledge. Figure 1 displays the linear prediction of skills and knowledge scores across gender with 95% CI. Figure 1A demonstrates that the skills and knowledge of YRI participants’ peers are significantly higher than control participants’ peers. Figure 1B shows that the skills and knowledge of female peers is significantly lower than that of male peers.

### 3.2. Qualitative Analyses

#### 3.2.1. Whom YRI Participants Shared with

In dyadic interviews and focus group discussions, YRI participants generally agreed that it was easiest to share with individuals who were the same age. For example, one YRI participant described how it was easiest to share with someone his own age: “*It’s my age mate that I would teach these skills … if we are the same age, or even if I’m older than her a little, I will notice that it’s good and she won’t take it as a bad thing*”. (Female, 27, Kailahun). There was one exception where one participant (Male, 30, Koinadugu) stated that he would prefer to share YRI skills with “*someone who is older*”.

A second common theme regarding who was easier to share with was related to the quality of relationships. Most YRI participants felt that it was easier to share skills with peers who they had close relationships with. One participant explained that “*If you are not friends sometimes you will see it that it is difficult to explain to them*”. (Male, 26, Kailahun). Another peer stated that “*[the YRI participant and I] had been joking before, so it was easier for him to share what he has learned … we have been acquainted for so long and that made me enjoy it so much as he was sharing with me*”. (Male, 32, Koinadugu).

Furthermore, two YRI participants described that gender was also an important factor when sharing skills and components and that it made them more comfortable to share with same-gender peers, for example: “*I can’t explain the skills to someone’s wife because he would think that I want her … you understand, right? So, I will forget about that*”. (Male, 30, Koinadugu).

#### 3.2.2. What YRI Participants Shared

The most commonly shared YRI skills and components were mindfulness-based emotion regulation strategies, specifically, “deep belly breathing” and “progressive muscle relaxation.” Nearly two-thirds of YRI participants shared progressive muscle relaxation and/or belly breathing with their peers. Participants wanted to share these skills because it helped them relax and feel calm when they were having interpersonal challenges or feeling stressed:

“*When I am in distress, I sit and do belly breathing, and as soon as I do it, I will calm down even if someone did something wrong to me. I will forget about anything thing that is affecting my life as soon as I do belly breathing*”.

(YRI participant in focus group 4).

YRI participants thought both of these skills were important to share with their peers because they prevented quarreling, helped keep moods calm, and helped people relax when they were stressed or tense about a situation:

“*My friend gets angry all the time and is hot-tempered. So, the only skill that I shared with him is belly breathing so that he could be calming down himself all the time. I told him ‘dude, when a crowd is gathered or a meeting called and we are in attendance, you will not get out from there without quarreling with someone, because you are hot tempered. If you are speaking and get stuck, just do belly breathing … just act as if you want to turn a bit and then give your back to the crowd and then do it quickly*’”.

(Male, 30, Koinadugu).

In addition to perceiving belly breathing as a useful skill, participants found that it was easy to share and easy for peers to learn. However, several youths believed that progressive muscle relaxation was more challenging:

“*For the progressive muscle relaxation, it would be a little bit difficult to do it. But for the belly breathing, she would sit down and do it … we were even telling her that she was doing belly breathing every time…she would say, ‘Let me do belly breathing, and she would do it. So, I came to realize that that is easy*’”.

(Female, 27, Kailahun).

Goal setting was another common YRI skill or component that youth shared with their peers. The metaphor of a ladder was used by facilitators to teach youth how to set Specific, Measurable, Achievable, Relevant, and Time-Bound (SMART) goals. Youth visualized each rung of a ladder as a small step towards achieving their larger goals. One YRI participant described the merit of sharing this with her peer:

“*We were given assignment to set our goals … for that, we were told to teach people when we return. So, when we would go back, we would explain to the people. When I would come and explain to people, sometimes it would make them happy… like my sister, she and her husband had wanted to get separated … during the training period. I (helped her) set the goal and I gave the steps as to how it could be solved*”.

(Female, 27, Kailahun).

Other components of the YRI that nearly all youth found to be useful were identifying positive coping strategies such as listening to music and talking with friends. A peer of a YRI participant described what she had learned from her friend: “*She said that if you are discouraged, you should try to mingle your children. When you are discouraged and your children are not around, you should play music, or you go outside*”. (Female, 30, Kailahun). One YRI participant would speak with her friend when she felt troubled, and through that experience, she would share what she had learned:

“*Sometimes I used to sit before and think that I needed someone to talk to. I would go inside and lie down and stay in alone. But through the training, I realized that it does not really make sense, and I started mingling with people … (my friend) will make sure to know if I am inside and she goes inside and starts to encourage by asking me what happened. There came a time that I was the one who started teaching her about the training, but now she has also started teaching me more than how I used to teach her. She started visiting me and we started doing things in common; and that is how I started forgetting about how I used to feel and I ended up forgetting about everything. So, we now play and laugh together*”.

(Female, 25, Kono).

#### 3.2.3. Comprehension of Skills

YRI participants described how perceived comprehension of skills could be a barrier or facilitator to sharing skills with their peers. One YRI participant describes how he had difficulty conceptualizing the SMART goals and the ladder technique; he found this problem-solving skill to be most difficult to understand in relation to other skills. He mentioned how his confusion stemmed from needing to change his behaviors and attitudes surrounding his goals to achieve success by advancing in the ladder:

“*I did not quickly understand the skill that has to do with the ladder from one to five. That one is difficult to understand. I understood all the remaining skills, but (the ladder) was not easy because it is not easy to jump from ladder one to ladder five. The one dealing with Mariatu (a Sierra Leonean parable) is easy because it is a story. The other one that has to do with behavior and characters, and it is difficult to understand*”.

(Male, 30, Koinadugu).

Additionally, one participant found one of the metaphors/activities related to cognitive restructuring (i.e., reframing negative self-perceptions more positively), called the “rock in the shoe activity”, more difficult to grasp. This participant struggled with properly understanding the metaphor used in the explanation of the relationship between thoughts, feelings, and behaviors. Another participant from a focus group explained how comprehension challenges caused him to stop trying to share this skill:

“*For me, the (rock in my shoe) was difficult, because even when people see me with a rock in my shoe, they will not understand what I am doing. So, because the people were not understanding it, I stopped doing it because I thought it was difficult*”.

(YRI Participant in Focus Group 4).

On the other hand, participants found the skill of using a visualization strategy involving a safe space or “safe place imagery” easier to understand and share. While it did not require them to perform a physical action, it is another strategy for relaxation and emotional regulation. One peer explained how he was able to understand the concept of safe place imagery when his friend explained it to him:

“*I think that the safe place imagery was simple, because you don’t perform any action—you go and sit at a quiet place with no noise. So, you will sit down and make up your mind by saying, ‘no, this is not good, this is good for me, this is not good for me.’ So, I think it’s very simple … it’s only you alone focusing your mind on something that is better for you. It was easier for me, because for all the others, you would have performed an action*”.

(Male, 25, Kono).

#### 3.2.4. How YRI Participants Shared

YRI participants used a variety of methods to share skills, including “explaining how the facilitators explained” (or verbal communication), demonstrating the use of the skill, and forming other small groups. Verbal face-to-face communication was most commonly used to explain the use of the skill and was sometimes performed in small, separate meetings with their peers. Some participants explained the different skills they learned and found useful through a storytelling process or a gradual progression from one stage to the next. One YRI participant explained how he shared with his friend:

“*Anytime they shared something new with us, I came home and called him … because they told us to teach our friends. So, for any day that I come back from the training, whatever question they gave us, I would explain to him … we do sit together and discuss the things they taught us in the training*”.

(Male, 29, Kono).

For other skills, some participants chose to use a physical demonstration of the skill while verbally explaining how to conduct the exercise. This approach was often used to share skills such as progressive muscle relaxation and belly breathing, where demonstrating what to do with their physical body in addition to verbal descriptions were used to relay the techniques. Many participants also mentioned using joking as another tool to facilitate sharing of skills they learned with their peers. One focus group participant talked about sharing with his friends: “*I teach them belly breathing and I will make jokes so that they will forget about the hurt*”. (YRI participant in focus group 3).

A few YRI participants also formed small groups with their peers to share the skills face-to-face, sometimes after a football match: “*When we go to the field, after the game, I call them all and explain the skills and what we learned. I always explain what I learned after the football match*”. (Male, 29, Koinadugu).

#### 3.2.5. Resulting Behavior Changes for Peers

Peers of YRI participants explained how their behavior and mental health changed as a result of integrating the shared YRI skills and components into their own lives and relationships. The most common change in externalizing behavior concerned reduced quarreling and fighting in intimate partner relationships. One peer described how his relationship with his wife improved, attributing this to his friend’s advice:

“*They (YRI participants) trained us, they talked to us. I have a wife and there was not a single day that we did not have a fight or quarrel. Now, (my friend) kept talking to me, and encouraging me, and I would never do it again. This is one of the changes that I experienced in my life. They advised us on how to relate with other people- our families- and how to care for the children*”.

(Male, 22, Kailahun).

Many peers reported they were “going out in the street,” joining gangs or going to clubs less after having YRI skills shared with them. These behaviors were perceived by both peers and YRI participants as detrimental to individuals. For example:

“*I was involved in all sorts of bad habits. I was going out with gangsters, and I have lived with them. They were doing bad things around the town. That time I was tattooing and I had the machine. Thank God that they taught my friend and he shared it with me too. My tattoo machine—I have destroyed it and thrown it away. I am happy that they taught my brother and he too wants to teach me. From that time until now, I have changed*”.

(Male, 22, Kailahun).

Several other peers provided examples of obeying elders after using YRI skills that were shared with them. One peer described the change in the way he related to elders:

“*When an elder would say something to me, the manner in which I would respond to him will make him feel bad, so we would quarrel … So, she came and explained all (the skills) to me and that has directed me about how to behave. So, I say thanks to God*”.

(Female, 29, Kono).

## 4. Discussion

The current study explored mechanisms of diffusion of the Youth Readiness Intervention (YRI), an evidence-based mental health intervention, across peer networks of youth in rural, post-conflict regions in Sierra Leone. Quantitative findings suggest a possible pathway of diffusion of YRI skills and knowledge from youth who participated in the YRI to their corresponding peers, as reflected in greater knowledge of YRI skills and components among peers of YRI participants compared with control participants. Qualitative findings on which YRI components were most often shared, how they were shared, and with whom they were shared help illuminate which YRI components are most transferable as well as how they might be most easily diffused to increase penetration of evidence-based mental health interventions and Sierra Leone and other post-conflict settings. Ultimately, this may help support the positive adjustment of a broader segment of youth residing in post-conflict settings.

Peers of YRI participants demonstrated significantly higher levels of knowledge about core YRI components than peers of control participants, suggesting that aspects of cognitive behavioral and interpersonal therapy techniques can be feasibly shared across social networks of youth living in rural, post-conflict settings with limited resources. Findings align with recent social network studies with adolescents, demonstrating that information on health interventions diffuses from participants to their corresponding peers [39,40]. In low-resource settings such as Sierra Leone, one way to maximize the benefit of evidence-based interventions could be by specifically suggesting that intervention participants “spread the word” and encourage their friends and family to apply skills learned from intervention participation. In addition, given that prior research on task-sharing approaches in Sub-Saharan Africa has shown that non-professionals, with appropriate training and support, can effectively deliver clinical interventions [41,42,43], training former YRI participants as YRI facilitators could help expand the reach of evidence-based mental health interventions and minimize financial costs. Future research should better disentangle which characteristics of intervention participants might be necessary to ensure competency and effectiveness of peer-to-peer transfer of skills.

In contrast to the study hypothesis that diffusion would be most likely to occur with same-gender peers, quantitative findings indicated that diffusion was not associated with any personal network characteristics of index participants. This might be related to the type of relationship between many index participants and peers, which was a spouse. Given that the age range of our sample was 18–30, and most participants had children and/or spouses or partners, YRI participants may have been more likely to share skills learned with their spouse and/or partner rather than a same-gender peer. Future research might systematically investigate the process of diffusion among romantic partners or household members (i.e., parent-to-child diffusion) in Sub-Saharan Africa. Additionally, quantitative and qualitative findings indicated that diffusion was more likely to occur between same-age peers and peers who were the emotionally closest. This provides preliminary support for a peer-led model and/or testing of specific strategies to encourage the peer-to-peer transfer of knowledge. Peer-to-peer support/counseling has emerged as an effective delivery strategy for youth with mental health problems in a variety of high- and low-income contexts [44,45,46,47,48]. If the process of sharing skills and information is perceived as offering mutual support from an “age mate” rather than lecturing or instructing from an elder, diffusion or sharing of skills may be more effective. Future research efforts might include testing strategies to encourage diffusion as well as exploring peer-to-peer delivery approaches, with consideration for developmental matching.

The sole method of sharing YRI components was via face-to-face individual or small group meetings. The most commonly shared YRI components were deep belly breathing and progressive muscle relaxation, in line with our hypothesis that some of the simplest-to-use components would be those that were easiest to share. In addition, several study participants noted sharing these skills because they allow for space to reduce potential conflicts and manage the tension that may lead to social disagreements. Compared to YRI components that involve cognitive strategies to improve emotion regulation, deep belly breathing and progressive muscle relaxation are both behavioral emotion regulation strategies, and both of these strategies have been shown to reduce psychological distress as well as physiological arousal levels [49,50,51]. Youth may experience more tangible, physiological benefits when using these strategies, which in turn may increase their satisfaction and/or preferences for these strategies and the likelihood that they will share them with others. For example, one YRI participant reported that they shared deep belly breathing with a friend that was often “hot-tempered’. Further adaptation and rollout of the YRI that leverages peer-to-peer transfer might consider focusing predominantly on teaching the simplest to use behavioral emotion regulation strategies that can be shared through demonstration, like deep belly breathing.

### Limitations

This study contains a few limitations. First, because participants resided in rural regions of an LMIC, findings might not translate to more urban regions or higher-income countries. Youth and families in rural regions might more frequently engage in communal behaviors, reciprocity, and sharing as part of everyday life due to the shared nature of agricultural work, collectivist culture, and the need for interdependence to ensure safety and stability. It is also possible that differences in proximity to peers and population density may impact peer-to-peer skill sharing. For instance, as seen in the bystander effect [52,53], deferral of responsibility occurs in more highly populated areas wherein individuals are less likely to offer support and aid to a neighbor in need because of the assumption that someone else will take on the responsibility. Our participants identified as male or female; study findings may not generalize to other populations that include people who do not identify as either male or female. Additionally, we did not collect baseline data on YRI skills and knowledge from index participants or their peers. This prevented us from determining whether increases in the level of YRI knowledge in index participants were associated with the level of knowledge in peers. Future studies should collect data prior to any intervention exposure among both intervention participants and their peers to assess knowledge gains better, as well as mechanisms of diffusion across networks over time. Investigating other socially based characteristics of intervention participants, such as popularity, social status, and social influence might shed further light on which intervention participants might be targeted as peer facilitators. Future studies might include a more specific assessment of concepts/constructs related to Rogers’ Diffusion of Innovations Theory, such as persuasion, to better understand if and how these constructs facilitated diffusion.

## 5. Conclusions

The current study used a mixed methods approach to investigate the naturalistic diffusion of an evidence-based mental health intervention among peer networks of youth in a rural, post-conflict, low-resource setting: Sierra Leone, West Africa. Building local knowledge and expertise in evidence-based mental health interventions among non-professionals residing in rural, low-resource communities may help address limitations in service access related to poor infrastructure, mobility related to agricultural work, poor network connectivity, and limited numbers of trained mental health professionals. It may also help promote resilience and positive adjustment of youth who have endured compounded adversity in post-conflict settings at a low cost. Findings contribute to significant gaps in the research on the diffusion of evidence-based mental health intervention among youth, particularly in Sub-Saharan Africa, and provide preliminary support for peer-led delivery models. Harnessing naturalistic processes of diffusion of innovations may ultimately increase the reach and penetration of evidence-based interventions, and developing tools to promote the diffusion of the most transferrable YRI components across peer networks could help maximize the benefits of evidence-based interventions that foster resilience, psychological adjustment, and overall mental health among youth in post-conflict, resource-constrained settings.

## Figures and Tables

**Table 1:** (**a**) Dyadic interview participants; (**b**) Focus group discussion participants.

(a)				
Dyad Interviews				
	Individual	Age	Gender	District
Dyad 1	YRI Participant	20	M	Kailahun
	Peer	22	M	Kailahun
Dyad 2	YRI Participant	27	F	Kailahun
	Peer	30	F	Kailahun
Dyad 3	YRI Participant	20	F	Kailahun
	Peer	62	F	Kailahun
Dyad 4	YRI Participant	26	M	Kailahun
	Peer	22	M	Kailahun
Dyad 5	YRI Participant	29	M	Kono
	Peer	22	M	Kono
Dyad 6	YRI Participant	25	F	Kono
	Peer	20	F	Kono
Dyad 7	YRI Participant	25	F	Kono
	Peer	25	F	Kono
Dyad 8	YRI Participant	29	M	Koinadugu
	Peer	32	M	Koinadugu
Dyad 9	YRI Participant	30	M	Koinadugu
	Peer	32	M	Koinadugu
Dyad 10	YRI Participant	20	F	Koinadugu
	Peer	25	F	Koinadugu
Dyad 11	YRI Participant	25	M	Kono
	Peer	23	M	Kono
**(b)**				
**Focus Group Discussions**				
**Group**	**District**	**Individual**	**Age**	**Gender**
Focus Group 1	Kailahun—Kpeje Bonge Chiefdom	YRI Participant	18	F
		YRI Participant	26	F
		YRI Participant	25	M
		YRI Participant	26	M
		Peer	40	M
		Peer	24	F
Focus Group 2	Kailahun—Penguia Chiefdom	YRI Participant	29	F
		YRI Participant	20	F
		YRI Participant	30	M
		YRI Participant	29	M
		Peer	20	F
		Peer	28	M
Focus Group 3	Kono—Nimikoro Cheifdom	YRI Participant	20	M
		YRI Participant	22	M
		YRI Participant	28	F
		YRI Participant	26	F
		Peer	22	F
		Peer	24	M
Focus Group 4	Koinadugu—Wara Wara Chiefdom	YRI Participant	27	M
		YRI Participant	30	M
		YRI Participant	23	F
		YRI Participant	25	F
		Peer	21	F
		Peer	20	M

**Table 2:** Linear regression models predicting YRI skills and knowledge among peers.

	Model 1 (N = 757)	Model 1 (N = 757)
	Coef. (Robust Std. Error)	Coef. (Robust Std. Error)
Study arm (ref: control) *YRI*	0.02 (0.01) *	0.02 (0.01) *
Gender of peers	−0.14 (0.01) ***	−0.13 (0.02) ***
Proportion homophily of gender		−0.00 (0.00)
Gender diversity in peers		0.02 (0.04)
Relationship diversity		−0.04 (0.03)
Diversity in residential distance		0.02 (0.05)
Educational status of index participants (ref: never attended school)		
*Primary*		−0.04 (0.02) *
*Junior Secondary*		−0.03 (0.02)
*Senior Secondary*		−0.01 (0.02)
Relationship status of index participants (ref: no partner) *With partner*		0.002 (0.01)
Gender of Index participants		−0.01 (0.02)
Age of peers		0.0001 (0.0005)
Age of Index participants		0.001 (0.001)

* *p* < 0.05, *** *p* < 0.001.

## Data Availability

The data sets used and analyzed in the current study can be made available upon reasonable request to the Principal Investigator.

## Supplementary Materials

The following supporting information can be downloaded at: https://www.mdpi.com/article/10.3390/ijerph20054059/s1, Table S1: YRI Session Modules.
