# Supplementary material for: Exploring Naturalistic Diffusion of an Evidence-Based Mental Health Intervention across Peer Networks of Youth in Sierra Leone
Source: Int J Environ Res Public Health. 2023 Feb 24;20(5):4059. doi: 10.3390/ijerph20054059 (PMC10002214; doi:10.3390/ijerph20054059)
Supplement: Supplementary file 1 [file ijerph-20-04059-s001.zip › ijerph-2139991-supplementary.pdf]

Supplementary Table S1. YRI Session Modules.

Module	Components
**1: Introductions and building group cohesion**	• Introduces the format and goals of the intervention<br>• Provides an opportunity to begin building trust, motivation for group participation, and group cohesion<br>• Participants identify personal goals that group participation will help them achieve
**2: Trauma psychoeducation**	• Psychoeducation on traumatic stress reactions in order to normalize this experience for group participants<br>• Participants learn about emotions, how to identify them and how this skill will help them reach their goals
**3. Understanding the link between beliefs, bodies and behaviors**	• Participants learn about the link between what you think, how it makes you feel, and how you behave<br>• Participants identify healthy and unhealthy coping behaviors and discuss the impact of coping skills on reaching their goals
**4. Taking control of your life**	• Introduces concept of emotion-regulation and coping skills as one way of taking charge of your life<br>• Practicing of coping strategies and recognizing things you can change and things you cannot<br>• Processing loss and teaching emotion regulation strategies: behavioral activation and deep belly breathing
**5. Relaxation and emotion regulation**	• Participants reflect upon what types of behaviors can alter their mood for the better<br>• Review of belly breathing and learn about guided imagery (safe spaces)<br>• Participants identify fun activities that can disrupt negative thinking
**6. Dealing with the past, things lost/things gained**	• Focus on normative reactions to life disruption and loss related to traumatic experiences and loss<br>• Participants reflect on things lost but also things gained in their lives
**7. Sequential problem solving**	• Participants learn how to approach decision-making using sequential (step-by-step problem-solving)<br>• Practicing of sequential problem solving

**9. Review of coping skills and problem solving**	• Participants review and practice skills learned thus far<br>• Participants learn another relaxation strategy: progressive muscle relaxation<br>• Participants discuss use of the skills to attain their goals.
**10. Addressing negative self-perceptions**	• Interactive exercises are used show how negative beliefs affect their emotions and their behaviors<br>• Participants learn strategies for changing their focus to the positive, including distraction by engaging in a fun or relaxing activity
**11. Review of skills and relapse prevention**	• Review of the skills learned in the group and planning for future challenges<br>• The group will think about challenges in the future, review their warning signs for when they begin to feel upset, and discuss the strategies they have learned to help themselves feel better
**12. Celebration and moving forward**	• Reinforcing the important concepts mastered throughout the group process<br>• Participants reflect on the information that they felt impacted them the most.<br>• Participants have an opportunity to express appreciation of the things they have learned<br>• Celebrations in the community are an optional component
